# Current Role of Radiation Therapy for Multiple Myeloma

**DOI:** 10.3389/fonc.2015.00040

**Published:** 2015-02-18

**Authors:** Giampaolo Talamo, Christopher Dimaio, Kamal K. S. Abbi, Manoj K. Pandey, Jozef Malysz, Michael H. Creer, Junjia Zhu, Muhammad A. Mir, John M. Varlotto

**Affiliations:** ^1^Penn State Milton S. Hershey Medical Center, Hershey, PA, USA

**Keywords:** multiple myeloma, radiation therapy, palliative therapy, pathologic fractures, stem cell collection

## Abstract

**Background:** Radiation therapy (RT) is a treatment modality traditionally used in patients with multiple myeloma (MM), but little is known regarding the role and effectiveness of RT in the era of novel agents, i.e., immunomodulatory drugs and proteasome inhibitors.

**Methods:** We retrospectively reviewed data from 449 consecutive MM patients seen at our institute in 2010–2012 to assess indications for RT as well as its effectiveness. Pain response was scored similarly to RTOG 0631 and used the Numerical Rating Pain Scale.

**Results:** Among 442 evaluable patients, 149 (34%) patients and 262 sites received RT. The most common indication for RT was palliation of bone pain (*n* = 109, 42%), followed by prevention/treatment of pathological fractures (*n* = 73, 28%), spinal cord compression (*n* = 26, 10%), and involvement of vital organs/extramedullary disease (*n* = 25, 10%). Of the 55 patients evaluable for pain relief, complete and partial responses were obtained in 76.4 and 7.2%, respectively. Prior RT did not significantly decrease the median number of peripheral blood stem cells collected for autologous transplant, even when prior RT was given to both the spine and pelvis. Inadequacy of stem cell collection for autologous stem cell transplant (ASCT) was not significantly different and it occurred in 9 and 15% of patients receiving no RT and spine/pelvic RT, respectively. None of the three cases of therapy-induced acute myelogenous leukemia/MDS occurred in the RT group.

**Conclusion:** Despite the introduction of novel effective agents in the treatment of MM, RT remains a major therapeutic component for the management in 34% of patients, and it effectively provides pain relief while not interfering with successful peripheral blood stem cell collection for ASCT.

## Introduction

Multiple myeloma (MM) is a rare cancer, representing 1% of all malignancies, with an annual incidence of approximately 4-5/100,000 (1). In the past, the traditional treatment of MM consisted of corticosteroids and conventional chemotherapy, with or without stem cell transplantation (SCT). However, systemic therapy was often inadequate, and previous studies have shown that the majority of MM patients -approximately two-thirds- required the use of radiation therapy (RT) during the course of the disease (2–4). The goal of RT is to deprive cancer cells of their multiplication potential by targeting their DNA and damaging it with irreparable double strand breaks, either by direct interaction or indirectly, after generation of free radicals. Neoplastic cells are killed by a variety of mechanisms, including apoptosis, mitotic catastrophe, necrosis, senescence, and autophagy, but the main cell-death mechanism following RT is considered apoptosis (5–7). RT was recognized as an effective anti-MM therapy as early as 1931, when it was found to ameliorate symptoms and, in certain cases, to provide a lasting disease control (8). Plasmacytomas are exquisitely radiosensitive neoplasms (9), and RT has a potentially curative effect for both solitary plasmacytoma of bone and extramedullary plasmacytomas (10, 11). However, the role of RT in the treatment of MM is only palliative. Traditional indications for RT in MM are pain control for large osteolytic lesions, prophylactic treatment of impending pathological fractures, post-fracture pain, spinal cord compression, and treatment of extramedullary disease (3, 12–14). Palliation of symptoms is a major goal of therapy in MM, because skeletal-related events (SREs), such as painful lytic lesions and pathologic fractures, represent major causes of morbidity in this cancer (15). In fact, MM patients often require potent analgesic drugs to control bone pain and improve their quality of life, and SREs may still develop despite a therapeutic response to effective systemic therapy (16, 17), due to the slow repair of osteolytic lesions. The efficacy of RT in palliating pain is very high, and several studies have reported a 75–100% range of pain control with a relatively short course of RT (2–4, 14, 18). Most MM patients achieve significant pain relief with a local RT dose of 3,000 cGy given in 10–15 fractions (14).

In the last few years, the introduction in clinical practice of several novel agents, i.e., the three immunomodulatory drugs (IMiDs) thalidomide, lenalidomide, and pomalidomide, and the two proteasome inhibitors (PIs) bortezomib and carfilzomib, has produced significant disease responses and survival advantage in MM patients, revolutionizing the therapy of MM in all phases of treatment, i.e., induction therapy, maintenance, and in the setting of relapsed/refractory disease (19). The role of RT in the era of the novel biological agents has not been adequately assessed. In this study, we retrospectively evaluated the current role of RT in the treatment of MM, with particular attention to indications, impact on survival, and effect on collection of blood stem cells for autologous SCT.

## Materials and Methods

After permission from our Institutional Review Board, we performed a systematic retrospective review of medical charts of 449 consecutive MM patients seen in 2010–2012 followed at our institute, either directly or as consultants for community oncologists. Because a range of radiation doses were given, all dosing regimens were converted into biologic effective doses using an alpha beta ratio = 10 for myeloma cells (20).

For assessing the pain response after RT, we measured pain levels on an 11-point scale 0, 1, 2, 3, 4, 5, 6, 7, 8, 9, 10, according to the Numerical Rating Pain Scale (NRPS) (21), both at baseline (within 1 week from the beginning of RT) and 3 months after RT (±1 week). We adopted the criteria used in the protocol RTOG 0631 (22): “complete pain relief”: pain score of 0 at 3 months post-treatment; “partial pain relief”: improvement of at least three points from the baseline NRPS (without increase in the level of narcotic pain medication); “stable response”: post-treatment pain score the same as or within two points of the baseline pain score (with no increase in narcotic pain medication); “progressive response”: post-treatment increase of at least three points from the baseline pain score.

Clinical characteristics of cases and controls were compared with the unpaired two-sample student’s *t*-test for numerical variables, and χ^2^ test for categorical variables (but if any expected frequency was <2 or if >20% of the expected frequencies were <5, we used the Fisher’s exact test). Survival curves were obtained with the Kaplan-Meier method, and compared with the log-rank test. We used the Cox proportional hazard regression model to analyze the effect of several risk factors on survival. All *p* values were two-tailed, and values <0.05 were considered significant. Overall survival (OS) was calculated from diagnosis to last follow-up or death. The time of diagnosis was defined as the day of the initial bone marrow biopsy. Statistical analysis was performed using the program SAS^®^ software, version 9.3 (SAS Institute, Cary, NC, USA).

## Results

In our original dataset of 449 consecutive MM patients, 14 patients received RT for reasons different from MM, i.e., cancers of the prostate (*n* = 4), lung (3), skin (3), breast (1), uterus (1), esophagus (1), and pituitary adenoma (1). Seven of these 14 patients received RT only for reasons other than MM, and they were excluded from the analysis. Of the remaining 442 patients, 293 (66%) did not receive RT for MM (Group A, “RT-naive”), and 149 patients (34%) did (Group B, “RT-treated”). Table 1 shows the characteristics of the two groups. A statistically significant difference was found between the two groups regarding age, but not sex and race. At the time of diagnosis, patients in group B were more likely to have non-secretory MM, lower infiltration of plasma cells in BM aspirate, early ISS stage, and osteolytic lesions on skeletal surveys. They were more likely to receive treatment with autologous SCT and IV bisphosphonates, either pamidronate or zoledronic acid.

**Table 1:** **Characteristics of 442 consecutive patients with multiple myeloma (MM), divided in two groups, depending on whether or not they received radiation therapy**.

	Group A (RT-naive) *n* = 293	Group B (RT-treated) *n* = 149	*p*
Age at diagnosis
Mean, years (range)	64.4 (37−92)	62.3 (21− 86)	** 0.043**
Sex, male	170 (58%)	83 (56%)	0.64
Race, Caucasian	249 (86%)	132 (89%)	0.30
Paraprotein
IgG, IgA	233 (80%)	98 (66%)	**<0.001**
κ or λ light chain	55 (19%)	39 (26%)	
Non-secretory/other	5 (2%)	12 (8%)	
Osteolytic lesions on X-rays	142 (57%)	103 (79%)	**<0.001**
ISS
Stage I	82∕241 (34%)	59∕112 (53%)	** 0.002**
Stage II	61∕241 (25%)	25∕112 (22%)	
Stage III	98∕241 (41%)	28∕112 (25%)	
Plasma cells in BM aspirate
Mean (±SD)	49% (±28%)	37% (±32%)	**<0.001**
High-risk cytogenetics^a^	59∕205 (29%)	22∕103 (21%)	0.16
Initial diagnosis of SP	0	12 (8%)	**<0.001**
Treatment
Thalidomide	89 (31%)	52 (35%)	0.33
Lenalidomide	212 (72%)	120 (81%)	0.06
Bortezomib	244 (84%)	132 (89%)	0.13
Pomalidomide/carfilzomib	17 (6%)	14 (9%)	0.16
Stem cell transplant	172 (59%)	104 (70%)	** 0.018**
Bisphosphonates IV	226 (83%)	139 (96%)	**<0.001**
Secondary malignancies^b^	11 (4%)	4 (3%)	0.78

*p levels < 0.05 are shown in bold font*.

*BM, bone marrow; ISS, International Staging System; IV, intravenous; SP, solitary plasmacytoma*.

*Percentages may not total 100 due to rounding*.

*^a^Defined as: hypodiploidy/complex karyotype or chromosome 13 abnormalities at metaphase cytogenetics, or translocations t(4;14), t(14;16), or del(17p) at FISH*.

*^b^Basocellular and squamocellular carcinomas of the skin are not included*.

**Figure 1:**
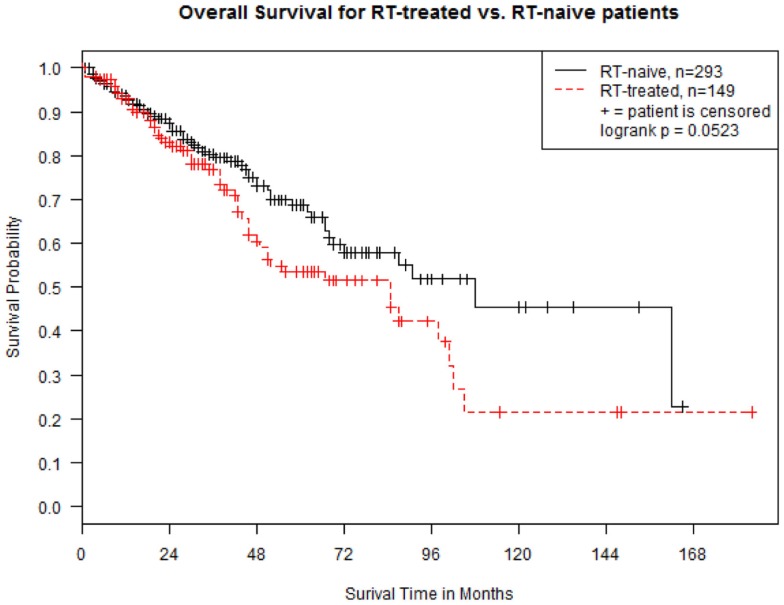
**Kaplan–Meier estimates of overall survival in 442 patients with multiple myeloma, divided in “RT-naive” (Group A, *n* = 293 -solid line) and “RT-treated” (Group B, *n* = 149 -dotted line)**. Survival is calculated from the time of diagnosis.

In both groups, median follow-up was 28 months (range, 0–184). Median OS was 108 months (95% CI 69–162) and 85 months (95% CI 49–98) in group A and B, respectively (*p* = 0.052 at Log-rank test; Figure 1). The OS between the two groups was re-examined in a multivariate Cox proportional hazard regression model by controlling for ISS stage (I, II, and III), cytogenetic status (high-risk vs. standard-risk), and presence of lytic lesions at skeletal survey. The results show that OS for group A patients is still significantly higher than that for group B patients (hazard ratio 0.43, 95% CI 0.21–0.72, *p* = 0.009). Hazard ratios were 0.59 (95% CI 0.28–1.05) for stage I vs. stage III (*p* = 0.10), 3.17 (95% CI 2.13–4.96) for high-risk cytogenetics (*p* < 0.001), and 1.35 (95% CI 0.86–1.59) for presence of lytic lesions at skeletal survey (*p* = 0.39).

Table 2 shows indications, sites of RT, and time to RT after the diagnosis of MM. A total of 262 sites were irradiated. The median dose of RT was 30 Gy (SD 7.4, range 0.8–50.0 Gy). The median number of fractions was 10 (SD 4.8, range 1–25), with 30 Gy given in 10 fraction being the most commonly used schedule (26.5% of RT courses). The most common indication for RT was palliation of pain, which accounted for 42% of all RT courses. A properly documented assessment of pain response according to the NRPS scale was available in only 55 patients: complete, partial, stable, and progressive responses were observed in 42 (76.4%), 4 (7.2%), 9 (16.4%), and 0 patients, respectively. The median biological effective dose (BED) was 37.5, 36, and 39 Gy in patients with complete response, partial response, and stable pain level, respectively, and ANOVA test found no significant dose/response relationship between BED and response (*p* = 0.76).

**Table 2:** **Indications, sites of treatment, and time to treatment of 262 radiotherapy courses in 149 patients with multiple myeloma**.

	No. of RT-treated sites	% of RT-treated sites^a^
**Indications for RT**
Palliation of bone pain	109	42
Spinal cord compression	26	10
Involvement of cranial nerves, neural foramina, cauda equina	17	6
Pathological fracture
Prophylaxis of impending fracture	33	13
Post-fracture RT	40	15
Involvement near vital organs/EMD	25	10
Esthetic reasons (painless bony protuberance)	12	5
**Sites for RT**
Skull/facial bones	26	10
Clavicle/scapula	20	8
Humerus/radius	20	8
Ribs/sternum	17	6
Spine
Cervical spine	16	6
Thoracic spine	47	18
Lumbar spine	32	12
Sacrum/pelvis	29	11
Femur/tibia	41	16
Soft tissues/EMD	14	5
**Time to RT**
Within 2 months of diagnosis	134	51
3–12 months from diagnosis	18	7
>1 year from diagnosis	110	42

*^a^Percentages may not total 100 due to rounding*.

*EMD, extramedullary disease*.

The second most common indication for RT were pathologic fractures (28%), either for their prevention before an impending fracture (13%), or as “consolidation” RT after surgery for the fracture (15%). Another frequent reason for RT were neurological complications (16%), such as spinal cord compression (10%), or impingement of cranial nerves, spinal neural foramina, or cauda equina syndrome (6%). Other less common indications included involvement of bones adjacent to vital organs (e.g., the sphenoid bone in the cranium; 10%), extramedullary disease (4%), and esthetic reasons (e.g., the development of a painless bony protuberance in the skull with deformity of the cranium; 5%). The most common site of RT was the spine (36%), and spine and pelvis constituted almost half of the radiated sites. In 51% of cases, RT was administered at the time of diagnosis of MM, either before or soon after (within 2 months) the initiation of systemic therapy. In 42% of cases, RT was given later during the course of the disease, more than 1 year after the initial diagnosis of MM. Only nine patients (6%) required a second course of RT for relapsed disease in a previously radiated site.

The median number of peripheral blood stem cells collected for autologous SCT, expressed as CD34^+^ cells × 10^6^/Kg, was 6.3 and 6.8 in group A (189 pts) and B (111 pts), respectively. The difference is not significant according to non-parametric Wilcoxon Rank-Sum *t*-test (*p* = 0.84). In group B, 48 pts underwent stem cell collection after RT to lumbar spine and pelvis, with a median dose of 45 Gy. Their median number of CD34+ cells × 10^6^/Kg collected was 5.2, and only seven of them (15%) failed stem cell collection. Seventeen of 189 patients (9%) in Group A failed stem cell collection (*p* = 0.41).

**Figure 2:**
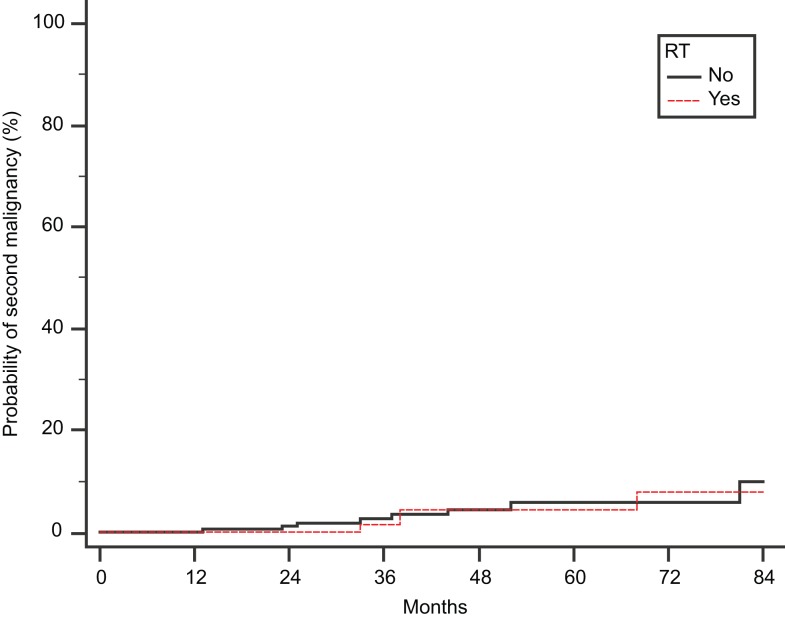
**Cumulative probability of second malignancy**. Time to develop a second malignancy is calculated from the initial diagnosis of MM.

Finally, we scrutinized our cohort of 442 patients for second malignancies, developed after the diagnosis of MM (Figure 2). We found 11 and 4 cases in Groups A and B, respectively (*p* = 0.78 by Fisher’s exact test). There were three cases of secondary acute myelogenous leukemia (AML), but they were not related to RT: these patients belonged to group A, and their AML was presumably attributed to the previous exposure to high-dose melphalan used as conditioning regimen for SCT.

## Discussion

Our study of 449 consecutive MM patients shows that RT continues to play a prominent role in the treatment of MM, despite the availability of effective novel agents, such as thalidomide, lenalidomide, pomalidomide, bortezomib, and carfilzomib. Approximately one-third of patients required the use of RT during the course of their disease. While RT can produce definitive cures in solitary plasmacytomas, its role in MM is only palliative. Although RT was associated with a survival decrement (Figure 1), we feel that this survival difference was due to patient selection. To our knowledge, only one study specifically assessed the effect of RT on the prognosis of MM patients, and no OS improvement was observed in a cohort of 162 patients (13), in accordance with our results.

The most common indication for RT in MM was pain palliation, and its efficacy in this setting was excellent: according to our data, a partial (7.2%) or complete (76.4%) response of pain to RT was achieved in 84% of patients, and no progressive pain/failure of RT was observed. Interestingly, the pain response seems to be more effective in this era of IMiDs and PIs, because previously complete and partial responses were reported in 21.6–30 and 69.8–71% of MM patients, respectively (2, 4, 14). Due to the retrospective nature of our study, we cannot be sure whether this improvement reflects only the benefit from RT, or the use of better types or higher doses of analgesics.

As expected, RT was mostly needed either at the time of diagnosis, when a clinical manifestation requires urgent intervention (e.g., spinal cord compression), or later during the course of the disease, when MM progresses. Its use is rarely required when the disease is in remission, during the induction chemotherapy. In fact, according to our data, only 7% of MM patients required RT between 2 and 12 months of initiation of systemic therapy, presumably due to the effectiveness of modern chemotherapy regimens. Initial diagnosis and refractory disease at progression are critical moments in the course of MM, because they may require RT for rapid local tumor reduction and “debulking” of focal lesions. The excellent activity of RT against MM has been known for decades. In 1971, Bergsagel observed that a single RT dose of 10 Gy can produce a 3 log cell kill, whereas drugs such as melphalan and prednisone produce <1 log cell kill (23). *In vitro* determinations of radiosensitivity in MM cell lines supported Bergsagel’s hypothesis (24, 25).

We found that the two most common indications for RT were pain palliation and pathological fractures, either for their prevention, or as “consolidation” therapy after surgery. This was expected, as RT is known to be an effective treatment modality in reducing the incidence of new fractures and focal lesions in irradiated bones of MM patients with vertebral lesions (18). In our series, other indications for RT included neurologic complications (such as spinal cord compression, cauda equina syndrome, and involvement of cranial nerves), extramedullary disease, and esthetical reasons, for example painless disfiguring bony prominences in the skull. Although spine and pelvis represented almost half of the RT sites, many other bones of both axial and appendicular skeleton were radiated, as shown in Table 2. In our patients, we never administered alkylating drugs (e.g., melphalan) and lenalidomide during the course of RT, because we feared their myelosuppressive effect. However, we have adopted the concomitant use of RT with corticosteroids, thalidomide, and bortezomib without significant problems (data not shown), when patients needed systemic cytoreduction at the same time of RT.

The baseline characteristics of our RT-treated and RT-naive groups were balanced for sex, race, and rates of high-risk cytogenetics. The statistical difference in age could theoretically be due to the fact that some very old patients have problems that prevent the administration of RT (e.g., poor performance status, comorbidities, dementia). ISS stage was significantly lower in the RT-treated group. This is probably due to a combination of factors: first, all MM patients who initially presented with a solitary plasmacytoma -clinically a more indolent plasma cell neoplasm than MM - received RT. Secondly, ISS stage III is defined by a serum level of beta-2 microglobulin >5.5 mg/dL, a finding that reflects not only high tumor load in the bones (which may need RT), but also renal insufficiency (which does not need RT). For reasons unknown to us, patients in the RT-treated group were more likely to have non-secretory disease. We can speculate that these patients come to medical attention for skeletal complications and require RT more frequently than patients with secretory MM, because the latter have useful tumor markers that may detect progressive disease at an earlier stage, or because they may present with MM manifestations that do not require RT, such as hyperviscosity syndrome due to IgG or IgA paraproteins, or cast nephropathy due to free light chain secretion (26). The association between RT and IV bisphosphonate use can be explained by the need for concomitant use of both treatments in a scenario of painful bone lesions and/or pathologic fractures. In fact, presence of osteolytic lesions at skeletal survey was more likely in the RT-treated group.

We found that a median dose of 30 Gy was administered for successful palliation. Studies done in the prior era of treatment reported that even lower doses, e.g., 10–20 Gy over 1–2 weeks, can provide adequate pain relief (2, 27). In the past, some investigations demonstrated worse response (14) or similar effectiveness with re-treatment (4). Since our data include only nine patients who required a second course of RT for relapsed disease in a previously radiated site, we cannot make meaningful conclusions on the palliative role of re-treatment. Of note, the significance of our findings regarding the palliative effect of RT in patients with MM is limited by two factors: the retrospective nature of our study, which predisposes it to a collection bias, and the limited number of evaluable cases: in fact, a properly documented assessment of pain response according to the NRPS scale was available in only 55 patients.

An important issue of RT in MM is its feasibility in transplant-eligible patients. Many MM experts avoid the use of RT - especially if directed to lumbar spine and pelvis – when they anticipate the need for collection of peripheral blood stem cells for autologous SCT, fearing toxicity of RT upon the hematopoietic stem cells. Published data on this subject remain controversial (28–30). In our analysis, RT did not induce a statistically significant reduction of the number of stem cells harvested, and it did not increase the number of patients who failed stem cell collection. Although we cannot make definitive conclusions, due to the retrospective nature of our study, it seems that palliative RT to the axial skeleton, if indicated, should not be denied based solely on fears of toxicity to the hematopoietic system.

Finally, regarding the development of secondary leukemias after RT, it is reassuring that we found no increased incidence of secondary malignancies among 149 RT-treated patients, albeit our median follow-up of 28 months is relatively short. Of note, all three cases of treatment-related AML observed in this time interval were found in patients who did not receive RT.

In conclusion, despite the use of novel effective systemic agents and SCT, RT continues to play a prominent role in the palliative treatment of MM. In our series, approximately one-third of patients required the use of RT during the course of their disease, most commonly for pain control and prophylaxis/treatment of fractures and neurological complications. Radiation provided effective pain control in greater than 80% of patients. Exposure to RT did not compromise the feasibility of peripheral blood SCT, and it did not seem to increase the frequency of secondary malignancies.

## Conflict of Interest Statement

The authors declare that the research was conducted in the absence of any commercial or financial relationships that could be construed as a potential conflict of interest.
